# Investigating the effect of remote ischaemic preconditioning on biomarkers of stress and injury-related signalling in patients having isolated coronary artery bypass grafting or aortic valve replacement using cardiopulmonary bypass: study protocol for a randomized controlled trial

**DOI:** 10.1186/s13063-015-0696-z

**Published:** 2015-04-23

**Authors:** Francesca Fiorentino, Gianni D Angelini, M-Saadeh Suleiman, Alima Rahman, Jon Anderson, Alan J Bryan, Lucy A Culliford, Marco Moscarelli, Prakash P Punjabi, Barnaby C Reeves

**Affiliations:** National Heart and Lung Institute, Cardiothoracic Surgery Department, Imperial College London, Du Cane Road, W12 0NN London, UK; Bristol Heart Institute, University of Bristol, Bristol Royal Infirmary, Marlborough Street, BS2 8HW Bristol, UK

**Keywords:** Coronary artery bypass graft surgery, Aortic valve surgery, Remote ischaemic preconditioning, Ischemia, Reperfusion, Randomised controlled clinical trial

## Abstract

**Background:**

Ischaemia-reperfusion injury occurs during heart surgery that uses cardiopulmonary bypass (CPB) and cardioplegic arrest. It is hypothesised that remote ischaemic preconditioning (RIPC) protects the heart against such injury. Despite the numerous studies investigating the protective effects of RIPC, there is still uncertainty about the interpretation of the findings as well as conflicting results between studies. The objective of this trial is to investigate the cardioprotective effect of RIPC in patients having coronary artery bypass grafting (CABG) or aortic valve replacement surgery. This will be achieved by estimating the effect of the intervention in the two groups of pathologies and by investigating the signalling mechanisms that may underpin the cardioprotective effect.

**Methods/Design:**

A two-centre randomised controlled trial will be used to investigate the effects of RIPC in two pathologies: patients having isolated CABG and those having aortic valve replacement surgery (AVR) with CPB. Participants will be randomised to RIPC or control (sham RIPC), stratified by surgical stratum. The intervention will be delivered by a research nurse. Data will be collected by a research nurse blinded to the intervention. The patient and the theatre staff are also blinded to the allocation. Markers of myocardial injury and inflammation will be measured in myocardial biopsies and in blood samples at different times.

**Discussion:**

This trial is designed to investigate whether RIPC will reduce myocardial injury and inflammation following heart surgery and whether there is a difference in effect between participants having CABG or AVR. This trial is a unique opportunity to study the mechanisms associated with RIPC using human myocardial tissue and blood, and to relate these to the extent of myocardial injury/protection.

**Trial registration:**

Current Controlled Trials ISRCTN33084113 (25 March 2013).

**Electronic supplementary material:**

The online version of this article (doi:10.1186/s13063-015-0696-z) contains supplementary material, which is available to authorized users.

## Background

### Remote ischaemic preconditioning and myocardial protection

Ischaemia-reperfusion injury occurs during heart surgery using cardiopulmonary bypass (CPB) and cardioplegic arrest. Cardioplegic arrest allows the surgeon to operate on a motionless heart. While the heart is arrested, there is no blood flow to the myocardium, which makes it ischaemic and can cause myocardial damage. When the blood circulation to the myocardium is restored at the end of cardioplegic arrest, reperfusion of the ischaemic myocardium induces further injury, often more severe.

Remote ischaemic preconditioning (RIPC) is a potentially protective phenomenon in which brief ischaemia of one organ or tissue (for example, arm or leg) is hypothesised to confer protection of another organ or tissue (for example, heart) against a sustained ischaemia-reperfusion insult [1-3]. Clinical benefits have been reported in patients prior to primary percutaneous coronary intervention (PCI) [4], in patients having paediatric [5,6] or adult open heart surgery [7-14] and during surgical repair of abdominal aortic aneurysm [15]. However, not all studies of RIPC have reported benefits [2,16,17].

A key challenge in interpreting the variation in findings has been a lack of evidence about mechanisms underpinning the effects of RIPC. In the absence of such evidence, it is difficult to know what other aspects of the treatment of patients (for example, choice of anaesthetic [18] and method of myocardial protection) may facilitate or inhibit the effect of RIPC and what aspects of the preconditioning stimulus are critical (for example, number and duration of cycles of ischaemia, timing in relation to onset of myocardial ischaemia, and applying the stimulus after rather than before sternotomy). It has been suggested that a sensation of pain (nociceptive preconditioning) may also be important [19,20].

The study population is another factor that may affect the effect of RIPC. For example, it has been suggested that the hearts of patients with ischaemic heart disease needing coronary artery bypass graft (CABG) surgery may already be pre-conditioned by virtue of the disease. This view leads to the hypothesis that the effects of RIPC should be greater in patients without ischaemic heart disease but still undergoing CPB and cardioplegic arrest, for example, patients having heart valve surgery [12-14]. Nevertheless, the evidence that has accrued to date from these proof-of-concept clinical studies in adult cardiac surgery has led to a large multicentre randomized clinical trial in patients having coronary artery bypass graft (CABG) surgery with or without valve surgery [21].

### Possible mechanisms underlying cardioprotection by remote ischaemic preconditioning

Although the mechanism by which a preconditioning stimulus in a patient’s limb confers protection to the heart is unknown [22], the preconditioned limb is presumed to transmit a signal to the heart, which, in turn, is presumed to trigger changes in the myocardium that eventually result in protection. Possible mechanisms have been proposed based on experimental models [1,23]. For example, it has been suggested that the ‘signalling’ mechanism between the remotely preconditioned organ/tissue and the heart could involve humoral and neural factors [24] or metabolites (for example, adenosine, bradykinin and opioids) [25-29]. More recent evidence suggests the involvement of hydrophobic mediators acting via PI3K/Akt-dependent pro-survival signalling [30]. There is also evidence that RIPC triggers an inflammatory response [6].

Similarly, little is known about the changes in the target tissue (myocardium) that are brought about by RIPC *before* ischemia and reperfusion. Most studies that have investigated changes in the myocardium have done so *after* reperfusion. It has been suggested that a signalling factor triggers intracellular signal transduction mechanisms in the heart, which are similar to those involved in cardiac ischaemic pre- and post-conditioning [1]. These could include activation of pro-survival kinases of the reperfusion injury salvage kinase (RISK) pathway, which could protect the heart by inhibition of the mitochondrial permeability transition pore (MPTP) [1]. It is possible that a variety of other signalling pathways involved in MPTP regulation [31] could also be involved in RIPC-induced protection. A recent experimental study [32] investigated the differential role of mitogen-activated protein kinase pathways, which suggested that the phosphorylation of p38MAPK, Erk1/2 and JNK could be involved in RIPC. Experimental evidence supports involvement of the mitochondrial Ca^2+^-activated K^+^ channel [33]. A very recent observational clinical study reported that RIPC was associated with changes in the phosphorylation of the signal transducer and activator of transcription 5 (STAT5) [11]. Most of these studies point to the mitochondria as a potential target for inducing protection by RIPC. We hypothesise that kinases involved in regulating the permeability of the MPTP are the mediators of RIPC-induced cardioprotection. Therefore, these enzymes and related signalling pathways will be monitored to identify the main pathway(s) responsible.

A key difference that is likely to exist between RIPC and cardiac pre- and post-conditioning stimuli (where the heart is made ischaemic for short periods) is related to changes in energy rich phosphates. Thus, RIPC is not expected to trigger cardiac changes associated with anaerobic metabolism (for example, drop in ATP, and build-up of ATP catabolites and lactate). However, a recent experimental study in mice has demonstrated that RIPC is associated with cardiac ischaemic stress and accumulation of cardiac adenosine prior to index ischaemia [34]. This highlights the need to measure ischaemic-stress related metabolites in the hearts of patients undergoing surgery using RIPC.

## Methods/Design

### Aims and objectives

The overall aim of the trial is to investigate the cardioprotective effect of RIPC in patients having CABG or aortic valve replacement surgery. Specific objectives are:

1. To estimate the difference in myocardial injury following CPB and cardioplegic arrest in groups of participants exposed to RIPC or sham RIPC.
2. To test whether the effect of RIPC versus sham RIPC differs in participants having CABG compared to participants having aortic valve replacement.
3. To investigate a range of possible signalling mechanisms that may underpin a cardioprotective effect.

No RIPC represents standard clinical practice in the two centres.

### Study design

**Figure 1:**
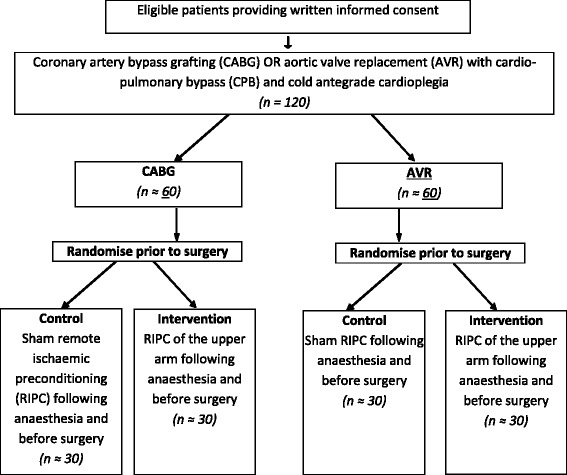
Trial schema.

This study is a two-centre randomised controlled trial investigating the effects of RIPC in two pathologies: patients having isolated CABG with CPB and those having aortic valve replacement surgery (AVR) with CPB. Participants will be randomised to RIPC or control (sham RIPC), stratified by surgical stratum. The randomization will be carried out by a designated member of the research team. The research nurse carrying out the intervention in the theatre and collecting the intra-operative and post-operative samples will not be involved in the data collection (see below). Another research nurse blinded to the allocation will do the post-operative data collection. Other trial personnel, participants and clinicians will be blinded to a participant’s random allocation. Figure 1 outlines the flow of trial participants in the study.

### Research approval

A favourable research ethics opinion has been granted by the Harrow NRES Research Ethics Committee London (reference 12/LO/1361).

### Study population and recruitment procedure

All patients referred for elective or urgent open-heart surgery with CPB will form the target population and be screened for trial eligibility. Male and female patients, aged ≥18 years, undergoing elective (or urgent) first-time CABG or AVR and who are willing to be followed-up and provide written informed consent will be eligible. Patients who have experienced cardiogenic shock or cardiac arrest, suffer from significant peripheral arterial disease affecting the upper limbs or have neither upper limb available for the intervention, have pre-operative renal failure (with a GFR <30 ml/min/1.73 m2) or have been taking glibenclamide or nicorandil within 24 hours of surgery will be considered ineligible. Patients who are participating in other interventional studies are also ineligible. These exclusion criteria are similar to the ones used by other researchers who have investigated the effects of RIPC [8,10].

All potential participants will be sent or given an invitation letter and Patient Information Sheet (PIS) (approved by the local Research Ethics Committee (REC)) describing the study. The patient will have time to read the PIS and to discuss their participation with others outside the research team (for example, relatives or friends) if they wish. Most patients will have at least 24 hours to consider whether to participate or not. In a few cases, this time interval may be as little as 12 hours or less, for example, for those patients admitted for urgent surgery without prior notification to the waiting list co-ordinator. Despite the short notice, it is important to include these patients for the applicability of the trial findings since about 40% of patients having cardiac surgery are admitted as urgent cases. Such exceptions were considered to be justified by the REC.

At the pre-assessment clinic or after admission to the cardiac unit for their operation, patients will be seen by a member of the local research team (study clinician/research nurse) who will answer any questions, confirm the patient’s eligibility and take written informed consent if the patient decides to participate. A copy of the consent form is provided in the Additional file 1.

A surgeon can decide not to randomise an otherwise eligible patient but has to give a reason for doing so. The trial screening log documents the reasons for ineligibility and, where provided, the reasons why an eligible patient declines to consent.

### Randomisation

Random allocations, stratified by surgical stratum and centre, will be generated by computer. Random treatment allocations will be (a) blocked with varying block sizes to ensure approximate balance in the number of participants allocated to each group; (b) generated in advance of starting the study; and (c) accessed using a secure, internet-based randomization system to guarantee concealment until a participant’s identity and eligibility is confirmed and securely documented. Randomization will be carried out after consent has been collected and before the operation by a designated member of the research team who will not be involved in data collection.

Participants will be blinded because the intervention is delivered in theatre and there will be no visible signs of having had RIPC. The operating staff and data collectors will be blinded because a research nurse, who will have no further role in the trial, will deliver RIPC or sham RIPC (see below).

### Trial interventions

#### Remote ischaemic preconditioning (intervention)

A research nurse will apply the cuff for administering RIPC under the operating cover, placing the cuff around the participant’s upper arm, after induction of anaesthesia and before sternotomy, CPB and cardioplegic arrest. RIPC will be induced as described by others [8,10]. It will comprise four 5 minute cycles of upper limb ischaemia, induced by a blood pressure cuff inflated to 200 mmHg, with an intervening 5 minutes of reperfusion by deflating the cuff. For participants with systolic blood pressure >185 mmHg, the cuff will be inflated at least 15 mmHg above the participant’s systolic blood pressure.

#### Sham remote ischaemic preconditioning (comparator)

A research nurse will position the cuff for administering RIPC as for the intervention group, under the operating cover and will squeeze the bulb with the air valve opened, at the same intervals as in the intervention group. This will ensure that all operating staff other than the research nurse placing the cuff will perceive the cuff to be inflated for all participants.

Both groups will have anaesthesia, sternotomy, CPB and cardioplegic arrest applied in accordance with a standard protocol. All patients received cold blood cardioplegia.

### Complications of intervention

The only possible complication attributable to the intervention is the onset of skin petechiae caused by cuff inflation. In this circumstance, a participant’s allocation is likely to become unblinded.

### Study centres

This study is a two-centre, randomised controlled trial. The two centres taking part are the Imperial College Healthcare NHS Trust (ICHT) and University Hospitals Bristol NHS Foundation Trust (UHBristol).

### Quality control of intervention and study procedures

A manual will be developed describing in detail all aspects of recruitment, randomization, interventions, data collection pertaining to blood and tissue sample collection and CRF completion together with safety reporting. This manual will be utilised to monitor adherence to procedural steps. The times of each cuff inflation and deflation will be recorded to monitor adherence to the intended intervention.

### Primary outcome

The primary outcome will be myocardial injury, assessed by measuring myocardial Troponin I in serum from blood samples.

### Secondary outcomes

The secondary outcomes will be obtained from data collected from myocardial biopsies and blood samples.

#### Markers in myocardial biopsies

The degree of ischaemic stress (anaerobic metabolism) associated with RIPC and cardioplegic arrest will be determined in ventricular biopsies. The metabolites that will be investigated in the ventricular biopsies are creatine phosphate, ATP, ADP, AMP, IMP, inosine, xanthine, hypoxanthine, adenosine, NAD^+^, lactate, alanine and glutamate. In addition, markers of potential signalling pathways that could be involved in RIPC will be monitored [23]. These secondary outcomes will be analysed in biopsies from the majority of participants, with a subgroup (about ten per group) being used for analyses of protein expression.

Expression of proteins implicated in RIPC protection in experimental studies will be determined by characterising the activation of intracellular kinases [35-37], the activation of the reperfusion injury salvage kinase (RISK) pathway, and the survivor activating factor enhancement (SAFE) pathway [38]. Gene expression for proteins that are activated will also be determined. Recent technological advances have provided new tools for screening and characterisation of activated proteins including mass spectrometry [39]. This is particularly important at the level of post-translational modifications of proteins (for example, phosphorylation), which are largely responsible for regulation of protein activity and function [40,41]. Therefore, the cardiac phosphoproteome will be measured in order to draw firm conclusions about the functional relevance of changes in proteins (proteome). A recent study has shown that RIPC cardioprotection in mice is linked with significant changes in several phosphoproteins associated with the z-disk area of the sarcomere [39]. Several of the phosphoproteins identified do not have antibodies available, thus making it difficult to measure using conventional methods. Therefore, we shall use these methods to screen for protein activation.

#### Markers in blood samples

Inflammation, systemic oxidative and metabolic stress associated with surgery will be assessed by measuring the plasma concentration of key biomarkers. These are 8-isoprostane, malondialdehyde (MDA) (markers of oxidative stress), IL-6, IL-8, IL-10, TNFα (markers of inflammation), and plasma lactate and blood pH (markers of anaerobic stress).

#### Other outcomes

Operative details and clinical outcome data will be collected for all participants. These will include aortic cross clamp and bypass time, ICU and hospital stay, inotropic support (quantity and duration), need for IABP, incidence of arrhythmia, myocardial infarction and routine measurements of serum creatinine to assess the impact of RIPC on renal function. Adverse events will also be recorded.

### Measurement of outcomes

#### Blood samples

Seven blood samples will be collected from each participant: two samples will be collected before the sternonotomy is performed and before CPB (one before RIPC and one after RIPC) is started, and five post-operative samples will be collected at 6, 12, 24, 48 and 72 hours after the end of ischaemic cardioplegic arrest. Each sample will amount to approximately 10 ml of blood stored in separate vials for plasma and serum analysis.

Serum concentration of Troponin I will be measured using automated assays. Plasma concentrations of IL-6, IL-8, IL-10 and TNFα will be measured using enzyme-linked immunosorbent assays (ELISA) or enzyme immunoassay (EIA) as described previously [42]. ATP catabolites, MDA, lactate and other relevant metabolites in plasma will be measured using HPLC and enzymatic kits as described above. In addition, plasma lactate and blood pH will be monitored at the same time points to determine the extent of systemic metabolic stress.

#### Ventricular biopsies

Four ventricular biopsies will be collected from each participant. Biopsies will be collected in pairs, using a Tru-cut™ needle to obtain one biopsy from the apex of the left ventricle and one biopsy from the anterior wall of the right ventricle; the biopsies will be ‘snap’ frozen in liquid nitrogen and transferred to -80°C until processing. The first pair of biopsies will be collected after harvesting the mammary artery and before starting CPB. The second pair of biopsies will be collected 20 minutes after the end of ischaemic cardioplegic arrest.

Metabolites will be measured using HPLC or commercially available kits with the standard protocols established in our laboratories [43,44]. Western blotting will be used to determine the effect of RIPC and cardioplegic arrest on the activation of key proteins involved in the regulation of the MPTP (that is, ERK1/2, GSK3β, Akt). Signalling pathways linked to these enzymes will also be monitored. Appropriate antibodies will be used to detect phosphorylated or total amounts of the proteins [31,32,45]. In addition to protein expression, mRNA for all relevant proteins will be determined, and the results validated [45]. The cardiac proteome and phospho-proteome will be studied using isobaric tandem mass tagging and analysed by reverse phase nano-liquid chromatography/mass spectrometry.

The schedule of data collection is outlined in Table 1.

**Table 1:** **Data collection**

	**Two ventricular biopsies**	**Blood samples**	**Participant’s characteristics**
Pre-op			✓
before RIPC		✓	
Immediately after RIPC		✓	
Immediately before CPB	✓		
20 minutes after the end of ischemic cardioplegic arrest	✓		
6 hr postop*		✓	
12 hr postop*		✓	
24 hr postop*		✓	
48 hr postop*		✓	
72 hr postop*		✓	

*postop = end of ischemic cardioplegic arrest. CPB, cardiopulmonary bypass; RIPC, remote ischaemic preconditioning.

### Data handling and storage

#### Data handling

Data will be entered onto a database designed specifically for the purpose, and data validation and cleaning will be carried out throughout the trial. Standard operating procedures (SOPs) for database use, data validation and data cleaning will be available and regularly maintained.

Trial data will be submitted directly into the database, which will be accessed via the NHS portal.

#### Data storage

All study documentation will be retained in a secure location during the conduct of the study and for 10 years after the end of the study, when all patient identifiable paper records will be destroyed by confidential means. Prior to destruction, paper records will be scanned and stored on the NHS server with limited password controlled access. Where trial-related information is documented in the medical records, these records will be identified by a label bearing the name and duration of the trial. Relevant ‘meta’-data about the trial and the full dataset, but without any participant identifiers other than the unique participant identifier, will be held indefinitely (Imperial College London server). A secure electronic ‘key’ with a unique participant identifier, and key personal identifiers (for example, name, date of birth and NHS number) will also be held indefinitely, but in a separate file and in a physically different location (NHS hospital server).

### Participant follow-up

Participants will be followed-up three months after their operation. They will be sent a questionnaire to capture the occurrence of any serious adverse events. Information about any hospitalization will be requested from the participant’s GP or the admitting hospital. Active participation in the trial terminates once the participant completes the follow-up questionnaire and sends it back to the research team.

### Sample size

Cardiac Troponin I is measured at the start of the operation (before RIPC or sham RIPC), after applying RIPC or sham RIPC, and five times after the end of ischaemic cardioplegic arrest and CPB. Determining an appropriate sample size requires several parameters to be specified. We have estimated some parameters from our previous research: average pre-post correlation = 0.3 and average post-post correlation = 0.5. We propose to recruit 30 patients/group in each surgical stratum, giving a sample size of 60 per surgical stratum and a total of 120. This sample size for each stratum is similar to that used in a previous trial in which RIPC was observed to have induced significant cardio-protection during CABG surgery [8]. Assuming an analysis of variance and covariance, this sample size will have 90% power to detect a standardised difference in serum markers of 0.43 between groups providing that there is no interaction between intervention/control and pathology. If there is an interaction, the sample size of 60 for each pathology will have 80% power to detect a standardised difference in serum markers of 0.55 between groups within each pathology stratum.

Assuming again an analysis of variance and covariance but without repeated measures, the sample size of 60 per group (assuming no interaction between intervention/control and pathology) will have 90% power to detect a standardised difference in cellular markers of 0.6 between groups. If there is an interaction, the sample size of 60 will have 80% power to detect a standardised difference in cellular markers of 0.72 between groups within each pathology stratum. Based on our previous research analysing myocardial biopsies, differences of this magnitude are plausible [46-49].

### Statistical analyses

#### Plan of analysis

Continuous outcomes will be summarised and presented graphically as geometric means and standard errors, assuming that a natural logarithmic transformation will be applied to the data to normalise their distributions [50]. The primary outcome, Troponin-I, and the secondary outcomes such as markers of kinase activity, oxidative stress and inflammation, are all measured multiple times in each patient (see Table 1). Therefore, Troponin-I and the other markers will be analysed by fitting multilevel mixed effect linear regression models, which consider the repeated measures in an appropriate and efficient manner. These models will fit both fixed effects (intervention, surgical stratum, centre, time and the interactions of intervention x time and intervention x centre) and a random effect. The fixed effects are analogous to standard regression coefficients and are estimated directly. The random effect is not directly estimated but is summarized according to its estimated variances and covariance. Baseline (pre-intervention) measures, where available, will be analysed jointly with the post-intervention measure(s). The interaction of group and surgical stratum will be tested and, if statistically significant at the 10% level, the treatment effect will be reported for each stratum separately; otherwise, an overall treatment effect will be given. Similarly, interactions of group and time and group and centre, will be explored (overall or within stratum depending on the significance of the group by stratum interaction) and, if statistically significant at the 10% level, the treatment effect will be reported for each time point separately.

Analyses will be adjusted for stratification factors, namely surgical procedure and centre. Findings will be reported as effect sizes with 95% confidence intervals. Analyses will be based on the intention-to-treat; cross-overs are expected to be rare. The frequencies of complications and conversions will be tabulated descriptively, by pathology. However, the trial is not powered to detect differences in adverse events and no statistical comparisons will be carried out.

It is important to emphasise that analyses of all outcomes will adjust for baseline levels of outcomes. Therefore, although there will be heterogeneity between patients within groups, the analyses will take this heterogeneity into account by quantifying the effects of the design factors after removing variation attributable to individual participants. This approach extends to the analyses of the proteome/phosphoproteome since data will be available for biopsies from the same ventricle of the same participant before and after cardioplegic ischaemic arrest. In effect, this allows us to focus on detecting changes in the same tissue (left or right ventricle) in two different pathologies.

#### Subgroup analyses

No sub-group analyses are planned other than those described above.

#### Frequency of analyses

The primary analysis will take place when follow-up is complete for all recruited patients. No formal interim analysis is planned. Given the nature of the intervention, we do not propose to have a formal Data Monitoring and Safety Committee (DMSC). We will report unexpected serious adverse events and deaths to the trial Sponsor. In these reports, the data will be presented by group but the allocation will remain masked, unless we are requested to disclose allocation because of concern about safety.

### Changes to the protocol since first approved

Substantial and minor amendments have been made. Inclusion and exclusion criteria have been amended to include a wider age range of patients and to facilitate recruitment. The primary outcome was changed from Troponin T to Troponin I because the coordinating centre measures Troponin I routinely. The type of blood sample collection was clarified in terms of distinguishing between serum and plasma concentrations. Anaesthetic details were removed to allow the operating team to follow their local practice. A more complete list of expected adverse events was added. Novel proteomic analyses on biopsies collected in a subgroup of participants have been added to characterise potential mechanistic pathways for RIPC. The sample size was increased by 25% to allow the proteomic analyses to be carried out in a subset of participants and to allow the trial to detect a smaller target difference between groups within each surgery stratum separately. The current version of the protocol is version 3 dated 17 July 2014.

### Measures to reduce the risk of bias

The trial has been designed to minimise the risk of bias. The trial is randomised, with stratification by centre and surgical stratum, so it is at low risk of allocation bias/confounding. Randomisation is unlikely to be subverted because random allocations are only issued after participants’ identity and eligibility are confirmed and securely documented. Blocked allocation could increase the risk of subversion bias, but this risk is being minimised by using varying block size. In order to subvert the trial, one person would need to know all allocations (across all surgeons) up to a particular timepoint; since different research nurses administer the intervention for different participants, this scenario would require collusion between research nurses.

The trial is at some risk of attrition bias because patients can decide to withdraw after randomisation or the care team could decide to withdraw them. However, we will try to minimise the probability of attrition bias by randomising the patient at a time close to the intervention. In addition, patients may not complete the follow-up questionnaire after discharge. We do not anticipate that attrition will be different between groups.

The trial is at low risk of ascertainment bias (outcome assessment) because the outcomes are objective and the analysts measuring blood or cellular markers are blinded to the allocation. Blinding of patients, the clinical care staff and the research team collecting the data, minimises risk of performance and detection biases. Success of blinding of patients will be assessed in the follow-up questionnaire.

Risk of bias in analysing the trial data is also low because analyses will be done according to a pre-specified statistical analysis plan and with an intention-to-treat basis.

### Dissemination of findings

The findings will be disseminated by usual academic channels, that is, presentation at international meetings, as well as by peer-reviewed publications and through patient organisations and newsletters to patients, where available. As the study compares surgical techniques no commercially exploitable findings are anticipated.

## Discussion

### What the study will investigate

We hypothesise that RIPC will reduce myocardial injury and reduce inflammation following heart surgery and that this effect will be greater in participants having AVR than in those having CABG. The effects of RIPC have been investigated in several trials to date, but their results have been inconsistent. By measuring many markers in myocardial biopsies and in blood samples, at different times after administering RIPC or sham RIPC, we expect to be able to relate changes in potential signalling mechanisms to the extent of myocardial injury measured by troponin I.

### Study design

The study design is a two-centre, randomised controlled trial with patients (CABG or valve) being randomly assigned to receive RIPC or control (sham RIPC) just before the surgery. This study design follows the one used in ERICCA [19]. Before this study commenced, we had discussions with the ERICCA trial team, and we tried to have our study as a sub-study to ERICCA. This was not possible because of the need for substantial protocol and governance changes, which were difficult to implement in a large ongoing multicentre study. Nevertheless, we adopted the same intervention and comparator. Our study population, however, is different; ERICCA is recruiting patients having CABG (with or without valve surgery) with an additive Euroscore greater than or equal to 5, whereas we have deliberately recruited patients undergoing CABG only or AVR only (regardless of their Euroscore). These differences in the trial populations meant that both centres taking part in this trial have also recruited to ERICCA at the same time.

### Challenges in study procedures

It has been suggested that some drugs interact with remote ischaemic preconditioning. Propofol and Fluorane, two of the most commonly used drugs in anaesthetic practice, are two such drugs. Propofol may inhibit STAT-5 transcription [51], hence reducing the preconditioning effect, while Fluorane may enhance protection [52]. However, there is a lack of firm evidence to substantiate these potential effects on the ischaemic preconditioning pathway. Therefore, for consistency, we have opted for the strategy of the ERICCA trial, which does not specify an anaesthetic protocol and allows each centre to follow its own practice. The two centres recruiting to this study adhere to their own, well-documented centre-specific protocols, minimizing variability in the anaesthetic procedures used between patients at the same centre. Stratified randomization ensures that patients allocated to both the intervention and control group within a centre will undergo the same anaesthetic procedures. Moreover, we will be able (albeit with low statistical power) to describe the interaction between centre and intervention.

Despite being a two-centre study, this study will recruit CABG patients at only one centre (ICHT). This is because the cardioplegia protocol for this operation differs between the two centres (warm blood versus cold blood), and it is believed that this might influence selected outcomes. This is hindering the recruitment rate, together with the challenges in recruiting patients to the AVR group in both centres because AVR operations are less frequent.

The intervention is carried out primarily in the anaesthetic room. Blinding of the operating team and carrying out the intervention can be challenging at this stage of the study because it coincides with the pre-operative anaesthetic procedures. The time a patient spends in the anaesthetic room can vary, and on some occasions the intervention can be incomplete by the time the patient is ready for theatre. To prevent delay of surgery, the intervention is completed in theatre as the patient is being prepared for surgery before sternotomy.

In order to monitor the changes in biomarker outcomes, blood samples are collected at various time points during a participant’s hospital stay. Some of these blood sample collections are scheduled outside office working hours. Even though reminders about the need to collect these samples are ‘handed over’ to the clinical team in charge of the patient when shifts change, on some occasions sample collection may be missed due to lack of time and other priorities. Similarly, there may be instances in which the biopsies are not collected; we aim to minimise this risk by having a member of the research team present in the theatre to prompt the operator to take the biopsies.

## Trial status

The trial opened for recruitment in one centre (ICHT) in February 2013 and in the other centre (UH Bristol) in June 2013. Recruitment is ongoing.

## Additional file

Additional file 1:
**Patient consent form.** Form used to obtain consent from patients.

## Abbreviations

ADP: adenosine diphosphate
AMP: adenosine monophosphate
ATP: adenosine 5’ triphosphate
AVR: aortic valve replacement/repair
CABG: coronary artery bypass grafting
CPB: cardiopulmonary bypass
CRF: case report form
GFR: glomerular filtration rate
EIA: enzyme immunoassay
ELISA: enzyme-linked immunosorbent assay
Erk: extracellular signal-regulated kinases
IABP: intra-aortic ballon pump
ICHT: Imperial College Healthcare NHS Trust
ICU: intensive care unit
IL-6: interleukin-6
IL-8: interleukin-8
IL-10: interleukin-10
IMP: inosine 5’-monophosphate
JNK: c-Jun N-terminal kinases
MDA: malondialdehyde
MPTP: mitochondrial permeability transition pore
mRNA: messenger ribonucleic acid
NAD+: nicotinamide adenine dinucleotide
P38MAPK: P38 mitogen-activated protein kinases
PCI: percutaneous coronary intervention
PI3K: phosphoinositide 3-kinase inhibitor
PIS: patient information sheet
RIPC: remote ischaemic preconditioning
REC: research ethics committee
RISK: reperfusion injury salvage kinase
SAE: serious adverse event
STAT5: transcription 5
TNFαm: tumor factor necrosis α
UH Bristol: University Hospitals of Bristol NHS Foundation Trust
