# Supplementary material for: Investigating the effect of remote ischaemic preconditioning on biomarkers of stress and injury-related signalling in patients having isolated coronary artery bypass grafting or aortic valve replacement using cardiopulmonary bypass: study protocol for a randomized controlled trial
Source: Trials. 2015 Apr 23;16:181. doi: 10.1186/s13063-015-0696-z (PMC4425928; doi:10.1186/s13063-015-0696-z)
Supplement: Additional file 1: — Patient consent form. Form used to obtain consent from patients. [file 13063_2015_696_MOESM1_ESM.pdf]

Local Principal Investigator:
Mr Prakash Punjabi
NHLI Cardiothoracic Surgery
B Block BN2/25,
Hammersmith Hospital
London W12 0NN
Tel: 020 8383 2026

Conditioning the heart to protect it during cardiac surgery

Patient Consent Form

Patient's Full Name *(please print)*:………………………………………………………………..

Date of Birth *(dd/mm/yyyy)*: ☐☐ / ☐☐ / ☐☐☐☐

Patient Study ID: ☐☐ - ☐☐☐☐

Please initial box to confirm you agree to each statement

Initials

1. I have you read and understood the Patient Information Leaflet (dated ____/____/____, version _____) ☐
2. I have had an opportunity to ask questions and discuss this study. ☐
3. I have received satisfactory answers to all my questions. ☐
4. I have received enough information about the study. ☐
5. (name)__________________________ explained the study to me. ☐
6. I understand that I am free to withdraw from the study at any time without giving a reason and that withdrawing from the study will not affect my medical care or legal rights. ☐
7. I agree to my GP being informed that I am taking part in this study. ☐
8. I give permission for sections of my medical records and/or data/images, relevant to my taking part in the research, being looked at by individuals from the study research team, the regulatory authorities or the hospital trust overseeing the research. I give permission for these individuals to have access to my records and understand that strict confidentiality will be maintained. ☐

Local Principal Investigator:
Mr Prakash Punjabi
NHLI Cardiothoracic Surgery
B Block BN2/25,
Hammersmith Hospital
London W12 0NN
Tel: 020 8383 2026

9. I agree to having my clinical information that may be held in this Trust, or in a national population register, being accessed by the clinical research team. ☐

10. I give permission for the study team to store my personal contact details and personal data on NHS computers at the study coordinating centre at Imperial College Healthcare NHS Trust. ☐

11. I give permission for the study team to retain and make further use of my data/samples that have already been obtained, if I withdraw from the study. ☐

12. I give permission for the study team to retain and make further use of my data/samples that have already been obtained and continue collecting the data for the study if I suffer a complication following the surgery which results in loss of capacity. ☐

13. I agree to take part in this study. ☐

______________________	______________________	______________
Name of patient	Signature	Date
______________________	______________________	______________
Name of person taking consent	Signature	Date

Original kept by research team, 1 copy for patient; 1 to be kept with hospital notes.

innovation respect care achievement pride
